# Potentially Severe Trauma Brain Injury Patients in a Non Reference Centre of Trauma Patients. Features and Evolution

**DOI:** 10.1186/2197-425X-3-S1-A491

**Published:** 2015-10-01

**Authors:** VA Hortigüela Martín, A Vidal González, H Pérez Martínez, V Trasmonte Martínez, C Pérez Calvo, M Pérez Márquez, A Gamo de Maeyer

**Affiliations:** Fundación Jiménez Díaz, Madrid, Spain; Hospital Rey Juan Carlos I, Móstoles, Spain; Hospital General de Villalba, Villalba, Spain

## Objectives

To analyze the patient profile, mechanism of injury, pre and post-hospitalization management and evolution of trauma brain injury (TBI) patients in a non-Intensive Critical Unit reference centre.

## Methods

A retrospective, descriptive study of all the TBI patients admitted in ICU of Fundación Jiménez Díaz of Madrid during the year 2014.

## Results

25 patients. Mean age 57 years. 22 (80%) males. 17 (68%) of the cases between October and Mars. Most frequent mechanisms of injury were: syncope 11 (44%), own height falling 9 (3%), high height falling 2 (8%). in 13 (52%) of the patients, intoxication signs were found, especially ethanol. 13 (52%) were picked up on the streets. the mean GCS (Glasgow Coma Scale) at pick up was 13.8, neurologic AIS (Abbreviated Injury Scale) 2.5 and ISS (Injury Severity Scale) 9. 18 of the cases (72%) were isolated head trauma cases. 12 of the patients (48%) were intubated at hospital admission. 1 emergency decompressive craniectomy was performed and 4 intracranial pressure sensors were placed. ICU average stay was of 4 days and the mean time on mechanical ventilation was 2 days. 1 tracheotomy was performed. the mean GCS at discharge was 12.8. Average GOS (Glasgow Outcome Scale) at ICU discharge, hospital discharge and after six months was 4, 4.5 and 4 respectively. Most common complications in the ICU were seizures, neurologic deficit and kidney injury. There was only 1 case of intracranial hypertension that evolved to encephalic death and became an organ donor. Patients that were picked up on the streets had fewer complications, intubations, length of hospital stay, time on mechanical ventilation and better GCS, but no differences in GOS were found.

## Conclusions

Although the complexity of the TBI patients that we receive is low, we manage a non-depreciable number of potentially severe trauma patients. the association between TBI and ethanol is important in our group. Patients picked up on the streets have better prognosis compared to the rest of our sample. So, and in spite of the existence of specialized trauma centres in our area, a good knowledge of this pathology with a correct management of these patients is necessary to detect complications and to obtain the best results.

